# Willingness to join community-based health insurance among rural households of Debub Bench District, Bench Maji Zone, Southwest Ethiopia

**DOI:** 10.1186/1471-2458-14-591

**Published:** 2014-06-11

**Authors:** Melaku Haile, Shimeles Ololo, Berhane Megersa

**Affiliations:** 1 Aman College of Health Sciences, Southern Nations, Nationalities and People’s Regional Health Bureau, P.O. Box 240, Mizan-Aman, Ethiopia; 2 Department of Health Services Management, Jimma University, Jimma, P.O. Box 371, Ethiopia; 3 Department of Monitoring and Evaluation, Jimma University, Jimma, P.O. Box 371, Ethiopia

**Keywords:** Community based health insurance, Willingness to join, Rural households, Southwest Ethiopia

## Abstract

**Background:**

Even though Ethiopia bears high burden of diseases, utilization of modern health care services is limited. One of the reasons for low utilization of healthcare services is the user-fee charges. Moving away from out-of-pocket charges for healthcare at the time of use is an important step towards averting the financial hardship associated with paying for health service. Prepaid plans for health are not accustomed in Ethiopia. Therefore, social and community based health insurance schemes were introduced since 2010.

In this study, willingness of rural households in Debub Bench District, to join community based health insurance was assessed.

**Method:**

Cross-sectional community based study was conducted in Debub Bench District in March 2013 using a pretested structured questionnaire. Two stage sampling technique was used to select 845 households as study units which were allocated to the kebeles proportionately. The sampled households were selected using simple random sampling technique**.** Data were entered into EPIDATA 3.0 and analyzed with SPSS version 20.

**Result:**

Among 845 sampled households, 808 were interviewed (95.6% response rate). About 78% of the respondents were willing to join the scheme. Most of demographic, socioeconomic variables and social capital were found to be significantly associated with willingness to join community based health insurance.

**Conclusion:**

If the scheme is initiated in the district, majority of the households will enroll in the community based health insurance. Farmers, the married households, Bench ethnic groups and illiterate, the dominant segments of the population, are more likely to enroll the schemes. Therefore initiation of the scheme is beneficial in the district.

## Background

In terms of access to modern health care and various other health indicators, Ethiopia ranks low even as compared to other low- income countries [1]. The country bears a high burden of disease mainly due to preventable diseases and conditions. For example, in 2011 all the top leading causes of mortality were easily preventable diseases such as malaria, pneumonia and respiratory tract diseases [2]. In spite of high burden, utilization of modern health care services is limited [3]. One of the reasons for low utilization of healthcare services is the user-fee charges [4].

In Ethiopia, 38.5% of the total health expenditure was covered through out-of-pocket charges, which is higher than that of other African countries, which was 30.6% in 2008 [3,5]. Nevertheless, Ethiopia’s per capita public spending for health (14 US$ in 2008) remains far below even that of other African and low income countries (83 US$ and 32 US$ respectively in 2008) [5].

Health care expenses were devastating and had long term effects on economic situations to the majority of households in Ethiopia. Consequently, it was suggested that alternative mechanisms such as health taxes should be established to cover health care expenses [6]. Moving away from out-of pocket charges for healthcare at the time of use is an important step towards averting the financial hardship associated with paying for health service [7]. But, in 2008 prepaid plans covered only 1.5% of the total private expenditure on health in Ethiopia [5].

To increase the prepaid plan coverage and access to modern health care services, Ethiopian government has developed health insurances strategy. Two types of health insurance schemes were introduced in Ethiopia since 2010. The first kind the schemes is social health insurance (SHI). Social health insurance is in implementation phase and intended to cover 10.46% of the population who are engaged in formal sectors. In Ethiopia, enrolling in SHI is compulsory for all in the formal sectors. This kind of health insurance scheme is expected to be fully implemented in the mid-of 2014.

The other health insurance scheme is community-based health insurance (CBHI), which is being piloted in 13 selected districts in Ethiopia and intended to cover 83.6% of the population of Ethiopia who are engaged in informal sectors; mainly those dwellers of rural areas [8]. The CBHIS has not been rolled out anywhere in the nation so far. It is expected to roll out after in 2015 or beyond throughout all districts in Ethiopia.

Unlike the SHI scheme, joining CBHI is based on voluntary decision of the households. In the pilot districts, households which join the community-based health insurance are expected to pay 180 Ethiopian Birr (10.4 US$) annually as a premium. However, the members’ contribution varies among the pilot districts ranging from 34.4 ETB-132 ETB [9].

Community based health insurance (CBHI) covers a wide variety of health insurance arrangements – with vast gradients in terms of ownership, management, membership, and service as well as financial coverage – in distinctive settings and designed for different population groups [10]. It is characterized by community-based social dynamics & risk pooling, solidarity, participatory decision-making & management, non-profitability & voluntary affiliation [11].

The benefits packages of CBHI in Ethiopia include all family health services and curative care that are part of the essential health package in Ethiopia when the scheme is scaled up to full implementation. Curative services include inpatient, outpatient services and acute illnesses [12].

Before establishing community-based health insurance scheme (CBHIS), its feasibility (i.e., its acceptance within the community and its sustainability) should be determined. Sustainability is determined by the design of the scheme, while acceptability must be tested in community surveys or in pilots through assessment of the people’s willingness to join (WTJ) & willingness to pay (WTP) before fully implementing CBHI [13].

In general demand studies are rarely collected or used as part of designing health insurance schemes in developing countries [14]. As a result, enrollments are low in many places where CBHI are established. For example a review of CBHIS found that 50% of them had less than 500 members while only 2% of the schemes had more than large number of enrollees (100,000 members) [15].

In Ethiopia, 76.4% people wanted to enroll in iddir based health insurance scheme by paying 7.60 Ethiopian Birr (ETB) monthly per household in 2007. In this study, monthly income, educational status and relation of respondent to household, participation in iddirs had statistically significant effect on willingness to pay for IBHIS [16]. Iddirs are funeral associations in Ethiopia that ensure a pay-out in cash and in kind at the time of a funeral for a deceased member of the family of a member of the group [17].

Another study elicited the community’s willingness to join and pay for a hypothetical community based health insurance scheme in rural Ethiopia using double bounded dichotomous choice contingent valuation method revealed that 60% of the rural people in Ethiopia were willing to join potential CBHI by paying 4.75 ETB (approximately, 0.60 US$) as monthly premium [18]. The study elicited the households WTJ and WTP for community-based insurance schemes by presenting community based health insurance scenario.

In the current study area, there are no any published data on demand of CBHI. Hence nothing is known about the level of acceptance of the CBHIS which is to be implemented nationally after two years. The investigators were initiated to conduct this study to fill this gap by assessing the WTJ the CBHIS in the specified area as it identifies the demand of the households for CBHI in the area. It is believed that this study will help policy makers to address factors which affect the households’ WTJ make the benefit of the planned community-based health insurance scheme.

The objective of this study was to assess willingness to join community-based health insurance scheme and factors associated with it among rural households in Debub Bench District of Bench Maji Zone, South-west Ethiopia, 2013.

## Methods

A community based cross-sectional study was conducted in rural kebeles of Debub Bench District, Bench Maji Zone; Southwest Ethiopia in March 2013. The district is one of the 9 districts in Bench Maji Zone of Southern Nations, Nationalities and People’s Region (SNNPR). The district is located 858 kms south west of Hawassa, the capital of SNNPR, and 588 kms south west of Addis Ababa, the capital of Ethiopia. There are 25 rural kebeles and 1 town administration in the district. The population of the district was projected to be 127,477 in 2012 [19]. (“Kebele” is the lowest administrative body in Ethiopia which comprises at least 1000 households or population of 5000 people).

**Figure 1:**
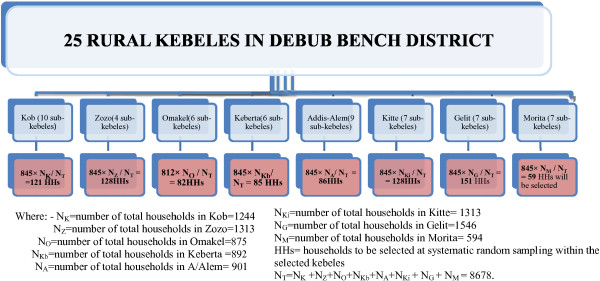
Schematic presentation of sampling procedure, Debub-Bench District, Bench-Maji Zone, 2013.

A sample size of 845 was calculated by a single proportion formula, taking P = 50% (expected rate of willingness to join community based health insurance scheme), considering a design effect of 2 and an anticipated non-response rate of 10%.A two-stage sampling technique was used to select participating households. The primary sampling units were kebeles. From 25 rural kebeles, 8 were selected randomly. The participating households were allocated to these 8 kebeles proportionate to the sizes of the kebeles and selected using simple random sampling technique (Figure 1).

The identity numbers of the houses, issued to each house in the kebeles by the administration of the district, were used to develop sampling frames of the households. Residents whose age were 18 years and above and who lived for more than six months in the kebeles were eligible for the study. Households with heads or spouses employed in formal sectors were excluded from the study because, according to the health insurance proclamation of Ethiopia, such households are covered by the social health insurance scheme which is in to be rolled out in the middle of 2014.

A pre-tested structured questionnaire was used. The questionnaire comprised of variables on demographic and socioeconomic characteristics, health status and health care and health related variables, social capital, participation in *iddirs,* willingness to participate in community based health insurance schemes and debriefing questions.

Before the respondent was asked his/her WTJ, community based health insurance scenario which was adapted from a willingness-to-pay study for CHI in Burkina Faso [20] and modified based on the benefit packages of Ethiopian community based insurance, was presented in details.

Presenting the scenario was important in this study since the respondents have little or no idea about community-based health insurance schemes. The scenario discloses the benefit packages of the scheme, membership payments and other issues about the CBHIS of Ethiopia (Additional file 1).

Data were collected by eight first cycle government school teachers. Two health professionals were recruited as field supervisors. Training was provided to both the data collectors and supervisors for two days and one day respectively. To reduce biases, the data collectors clearly told the respondents that they have no any link with the proposed CBHI.

Data were edited, cleaned, coded and entered in to Epidata 3.0, validated in double entry validation and exported to SPSS version 20.

The statistical analysis comprised three sequential steps. First, the associations between potential predictors of WTJ were assessed using contingency table analyses. The x^2^ statistic and its corresponding odds ratio, and 95% confidence intervals were computed to assess the significance and magnitude of these bivariate associations. To avoid unstable estimates, the cut-off point of 0.20 was used in the bivariate analysis to select variables for fitting a multivariate logistic regression model and identify the independent contribution of each variable while adjusting for the effects of other variables in the model.

The x^2^ statistic tests the overall statistical significance of the model, and adjusted odds ratios and their corresponding 95% confidence intervals were reported to assess the association between individual variables and willingness to join CBHI. Finally, it was evaluated that variables identified as associated (P < 0.05) with willingness to join the CBHI in the multivariate logistic regressing analysis were used to predict WTJ.

Ethical clearance was obtained from Ethical Review Committee of College of Public Health and Medical Sciences of Jimma University. Support letters were obtained from Debub Bench District Administration, education and health offices. Permission was sought from the Kebeles administration before conducting the study. Verbal consent was obtained from the respondents.

## Results and discussion

### Result

Of 845 sampled households, 808 participated in the study which yielded a response rate of 95.6%. The median age of respondents was 32, ranging 18–87 years. Among them, 574 (71%) were male, 550 (68.1%) Protestant Christians, 519 (64.2%) Bench ethnic groups, 675 (83.5%) married (574 monogamous/monandrous and 101 polygamous), 615 (76.1%) farmers, 388 (48%) were illiterate and 654 (80.9%) were head of the household. The median number of the household members was 5 with range of 1–13. The median annual household income, as estimated from the amount earned from sales of coffee, khat, maize cassava and other local products such as fruits, honey dairy products, etc., in one year time, was 2475 ETB (143 USD), ranging between 100–18,600 ETB (5.8-1075.1 USD). From the analysis of the wealth index, 31.6% of the households were found in the second and 24.0% in the highest wealth quintiles (Table 1).

**Table 1:** Demographic and socioeconomic characteristics of the study participants in Debub Bench District, Southwest Ethiopia, 2013 (n = 808)

**Description**		**Frequency (%)**
Sex of the respondent	Female	234 (29.0)
	Male	574 (71.0)
Relationship	Head	654 (80.9)
	Spouse	132 (16.3)
	Others (Child, parent)	22 (2.7)
Religion of the respondent	Protestant	550 (68.1)
	Orthodox	199 (24.6)
	Muslim	41 (5.1)
	Others	18 (2.2)
Marital status of the respondent	Monogamous/monandrous	573 (70.9)
	Polygamous/polyandrous	101 (12.5)
	Single	55 (6.8)
	Widowed	24 (3.0)
	Divorced	55 (6.8)
Occupation of the respondent	Farmer	615 (76.1)
	Housewife	113 (14.0)
	Merchant	36 (4.5)
	Student	30 (3.7)
	Others	14 (1.7)
Ethnicity of the respondent	Bench	519 (64.2)
	Amhara	116 (14.4)
	Kaffa	64 (7.9)
	Others	109 (13.5)
Educational status of the respondent	Illiterate	388 (48.0)
	Read and write	221 (27.4)
	Grade 1-8	178 (22.0)
	> = Secondary school	21 (2.6)
Wealth quintile of the household	Lowest wealth quintile	70 (8.7)
	Second wealth quintile	255 (31.6)
	Middle wealth quintile	164 (20.3)
	Fourth wealth quintile	125 (15.5)
	Highest wealth quintile	194 (24.0)
Category of annual income	Lower than 1100 birr	198 (24.5)
	1100-4300 birr	409 (50.6)
	More than 4300 birr	201 (24.9)

Seven hundred and forty seven (92.5%) of the households were participating in iddirs. Out of them 635 (85 were participating in one iddir) and the remaining 112 households in more than one iddirs. The median contribution of the households to iddirs was 1 ETB per month with range of 1–4 ETB.

Regarding individual level social capital, 233 (28.8%), 545 (56.4%) and 119 (14.7%) of the households were of low (lower than the 25th percentile), middle (between 25th and 75th percentiles) and high (above 75th percentiles of horizontal trust index) individual level horizontal trust respectively. Also, 222 (27.5%), 451 (55.8%) and 135 (16.7%) of the households were of low, middle and high individual level reciprocity respectively.

With respect to health status and health related variables, 50 (6.2%) of the respondents evaluated their family’s health status to be very poor and 98 (12.1%) very high. Sixty one (7.5%) of the participants had at least one member with chronic disease or disability; and 250 (30.9%) of the households had at least one member who had encountered illnesses 3 months prior to data collection. Among the ill 231 (92.4%) had sought treatment for the illnesses they experienced, and 219 (94.8%) got treatment. The remaining 12 did not get treatment because, mainly, lack of money (Table 2).

**Table 2:** Health and health related situations in Debub Bench District, Southwest Ethiopia, 2013

**Descriptions**		**Freq (%)**
Self-reported health status of the household (n = 808)	Very poor	50 (6.2)
	Poor	166 (20.5)
	Medium	270 (33.4)
	High	224 (27.7)
	Very high	98 (12.1)
Persons with chronic illness and/or disability in the household (n = 808)	No	747 (92.5)
	Yes	61 (7.5)
Any illness encountered during the past 3 mths (n = 808)	No	558 (69.1)
	Yes	250 (30.9)
Seek of medical treatment for the recent episode (n = 250)	No	19 (7.6)
	Yes	231 (92.4)
Get treatment (n = 231)	No	12 (5.2)
	Yes	219 (94.8)
Place of treatment (n = 219)	Private Heath Facility	90 (41.1)
	Public health center	65 (29.7)
	Public hospital	49 (22.4)
	Other (self-treatment, traditional healer and local drug vendor)	15 (6.8)
Reasons for going there (n = 219)	The HF was physically accessible	104 (47.5)
	The HF was not expensive	18 (8.2)
	The health facility not too crowded	19 (8.7)
	The health service was effective	66 (30.1)
	Other (specify)	12 (5.5)
Reasons for not getting treatment (n = 12)	No enough money	9 (75.0)
	Others (too far, self limiting)	3 (25.0)
Coverage of the health care cost (n = 219)	Self	204 (93.2)
	Others (free, community)	15 (6.8)
Satisfaction with health care service and costs (n = 219)	Very dissatisfied	23 (10.5)
	Dissatisfied	61 (27.9)
	Neutral	8 (3.7)
	Satisfied	111 (50.7)
	Very satisfied	16 (7.3)
Perceived quality of the health care service in the district (n = 219)	Very low	20 (9.1)
	Low	76 (34.7)
	Neutral	24 (11.0)
	High	87 (39.7)
	Very high	12 (5.5)
Concern of the household for covering health care costs (n = 219)	Very difficult	77 (35.2)
	Difficult	110 (50.2)
	Not difficult	32 (14.6)
Means of getting money for health care payment (n = 187)	Drew from the savings	38 (20.3)
	Borrow from someone	27 (14.4)
	Assisted by relatives	68 (36.4)
	Undertaken extra work	2 (1.1)
	Sell capital assets such as cows	33 (17.6)
	Cut back on other things, food, etc.	19 (10.2)
Borrow money for medical costs within last year (n = 808)	No	530 (65.6)
	Yes	278 (34.4)
The nearest conventional health institution to the respondents’ home (n = 808)	Health center	373 (46.2)
	Clinic (Private)	367 (45.4)
	Hospital (Gov)	68 (8.4)

Of 219 who got treatment, 41.1% preferred to go to private clinics. They preferred the specified institutions because of its physical accessibility (47.5%), effective service (30.1%), not too crowded (8.7%), not expensive services (8.2%), or other reasons (5.5%) (Table 2).

The median expenditure of the 219 households which sought treatments was 170 ETB with range of 18 to 2000 ETB. Two hundred and four (93.2%) of the households covered the medical expenses by themselves. One hundred and eighty seven (85.4%) of these households reported that it was (very) difficult to cover payments for treatments. As a result, 68 (36.4%) of them were assisted by relatives to cover the medical costs; 38 (20.3%) drew from their savings, and 27 (14.4%) borrowed from someone. The remaining had to sell capital assets such as cows (17.6%), cut back on other things, food, drink, cloth etc. (9.1%), undertook extra works and search for other means (2.2%) to cover the payments for treatment (Table 2).

Of 808 respondents, 278 (34.4%) reported that they had borrowed money for covering health care expenses within one year before the data were collected. The median amount that these households borrowed was 200 ETB (11.6 USD), ranging 30–2000 ETB (1.7-115.6 USD) (Table 2).

Regarding the distance of home of the household to the nearby health facility (private clinics, health centre or public hospital), it was reported that the median time it takes to reach the nearby health facility was 50 minutes, range between 3 minutes to 180 minutes (Table 2).

Among the participants, 629 (77.8%) were willing to join the proposed community based health insurance. Four hundred and seventy five (75.5%) of the respondents wanted to join the scheme to get free access to health care. And, 59 (33%) of 179 respondents did not want to join the scheme because they do not need health insurance (Table 3).

**Table 3:** Willingness to join community based health insurance, reasons for joining and not willing to join the scheme in Debub Bench District, Southwest Ethiopia, 2013

**Description**	**Frequency (percent)**
Willingness to join community based health insurance scheme (n = 808)	
Yes	729 (77.8)
No	179 (22.2)
Reasons for joining the schemes (n = 629)	
It provides free access to medical care	475 (75.5)
To help others	29 (4.6)
For security and peace of mind in times of ill-health	79 (12.6)
Facing health problem frequently	45 (7.2)
Other (specify)	1 (0.2)
Reasons for not joining the scheme (n = 179)	
I do not have enough money to pay	44 (24.6)
Do not need health insurance	59 (33.0)
Out-of pocket charge is better	17 (9.5)
Lack of trust in government programmes	8 (4.5)
Lack of functional HF in my village	24 (13.4)
H/insurance is a confusing scheme	14 (7.8)
Others	13 (7.3)

The study revealed that a number of variables affect the households’ decision in willingness to join the proposed community based health insurance scheme. In multivariate analyses, most of the demographic variables (age, relationship of the respondent to the household head, marital status, occupation and ethnicity of the respondent, as well as the household’s family size) were significantly associated with WTJ the CBHIS.

Age had negative associations with the probability of WTJ the CBHIS. The younger were 6% more likely to join the scheme than the older (95% CI of AOR: .914, .974). Spouses were 59% less likely to join the scheme, in comparison with heads of the households (95% CI of AOR: .174, .967). In comparison to monogamous/monandrous, the single were 87.7% less likely to join the scheme (95% CI of AOR: .032, .474). Occupationally, housewives were more likely to join the scheme than farmers (AOR = 11.917; 95% CI AOR: [4.017, 35.357]). Ethnically, households which belong to Kaffa ethnicity were 81.6% less likely to join the scheme than Bench (95% CI of AOR: .072, .468). Size of the family was positively associated with WTJ decisions of the households. As the number of the household members increase, the probability of WTJ increased by 69% (95% CI AOR: 1.363, 2.099) (Table 4).

**Table 4:** Factors which are associated with willingness to join community based health insurances in Debub Bench, 2013

**Variables**	**Freq (%)**	**WTJ**		**P-value**	**Crude OR**	**Adjusted OR [95% CI]**
		**Yes (%)**	**No (%)**			
Demographic variables						
Age		629 (78)	179 (22)	.000	1.035	.943 [.914, .974]
**Relationship**	808 (100)	629 (78)	179 (22)	.007		
Head*	654 (80.9)	519 (79)	135 (21)			
Spouse	132 (16.3)	97 (73)	35 (27)	.042	.721	.410 [.174, .967]
Others	22 (2.7)	13 (59)	9 (41)	.015	.376	18.523 [1.762, 194.6]
**Religion†**	808 (100)	629 (78)	179 (22)	.869		
Protestant*	550 (68.1)	431 (78)	119 (22)			
Orthodox	199 (24.6)	160 (80)	39 (20)	.936	1.133	.965 [.410, 2.275]
Muslim	41 (5.1)	24 (58)	17 (42)	.957	.390	1.042 [.233, 4.650]
Others	18 (2.2)	14 (78)	4 (22)	.406	.996	2.056 [.375, 11.262]
**Marital status**	808 (100)	629 (78)	179 (22)	.019		
Monogamous*	573 (70.9)	458 (80)	115 (20)			
Polygamous	101(12.5)	84 (83)	17 (17)	.061	1.241	.409 [.160, 1.043]
Single	55 (6.8)	25 (45)	30 (55)	.002	.209	.123 [.032, .474]
Widowers	24 (3.0)	19 (79)	5 (21)	.996	.954	.996 [.171, 5.814]
Divorced	55 (6.8)	43 (78)	12 (22)	.929	.900	.950 [.304, 2.971]
**Occupations**	808 (100)	629 (78)	179 (22)	.000		
Farmers*	615 (76.1)	501 (81)	114 (19)			
Housewives	113 (14.0)	87 (77)	26 (23)	.000	.761	11.917 [4.017, 35.357]
Merchants	36 (4.5)	19 (53)	17 (47)	.769	.254	.821 [.221, 3.046]
Students	30 (3.7)	14 (47)	16 (53)	.443	.199	.521 [.098, 2.760]
Others	14 (1.7)	8 (57)	6 (43)	.025	.303	.088 [.011, .738]
**Ethnicity**	808 (100)	629 (78)	179 (22)	.000		
Bench*	519 (64.2)	404 (78)	115 (22)			
Amhara	116 (14.4)	88 (76)	28 (24)	.341	.895	1.557 [.626, 3.875]
Kaffa	64 (8.0)	41 (64)	23 (36)	.000	.507	.184 [.072, .468]
Others	109 (13.5)	96 (88)	13 (12)	.004	2.102	5.306 [1.682, 16.733]
Total family size				.000		1.691 [1.363, 2.099]
Socioeconomic variables						
**Educational-status**	808 (100)	629 (78)	179 (22)	.001		
No education*	388 (48.0)	316 (81)	72 (19)			
Read & write only	221 (27.3)	176 (80)	45 (20)	.045	.891	2.134 [1.017, 4.479]
Grade 1-8	178 (22.0)	124 (70)	54 (30)	.007	.523	.321 [.140, .738]
Sec and above	21 (2.6)	13 (62)	8 (38)	.864	.370	1.205 [.143, 10.161]
**Wealth quintile**	808 (100)	629 (78)	179 (22)	.002		
Low wealth quintile	70 (8.7)	48 (69)	22 (31)	.451	.778	1.559 [.492, 4.938]
Second wealth quintile*	255 (31.6)	188 (74)	67 (26)			
Middle wealth quintile	164 (20.3)	124 (76)	40 (24)	.082	1.105	.481 [.211, 1.097]
Fourth wealth quintile	125 (15.5)	94 (75)	31 (25)	.375	1.081	.672 [.279, 1.618]
Highest wealth quintile	194 (24.0)	175 (90)	19 (10)	.003	3.282	4.203 [1.616,10.931]
**Annual income**	808 (100)	629 (78)	179 (22)	.003		
Less than 1100 birr	198 (24.5)	133 (67)	65 (33)	.008	.470	.475 [.274, .823]
1100-4300 birr*	409 (50.6)	326 (80)	83 (20)			
More than 4300 birr	201 (24.9)	170 (85)	31 (15)	.180	1.768	1.500 [.830, 2.712]
Participation in risk sharing organizations						
No. of risky to become ill†				.887	1.322	.980 [.780, 1.300]
**Iddir participation**	808 (100)	629 (78)	179 (22)			
Yes*	747 (92.5)	603 (81)	144 (19)			
No	61 (7.5)	26 (43)	35 (57)	.139		.427 [.138, 1.320]
Social capital						
**Indiv level hor trust**	808 (100)	629 (78)	179 (22)	.000		
Low	233 (28.8)	144 (62)	89 (38)	.000	.339	.064 [.025, .165]
Middle*	456 (56.4)	377 (83)	79 (17)			
High	119 (14.7)	108 (91)	11 (9)	.238	2.057	2.284 [.580, 9.000]
**Indiv level reciprocity**	808 (100)	629 (78)	179 (22)	.050		
Low	222 (27.5)	136 (61)	86 (39)	.952	.341	.975 [.435, 2.187]
Middle*	451 (55.8)	371 (82)	80 (18)			
High	135 (16.7)	122 (90)	13 (10)	.015	2.024	4.959 [1.362, 18.052]
**Commun level hor trust**	808 (100)	629 (78)	179 (22)			
High	158 (19.6)	146 (92)	12 (8)	.000	4.207	25.233 [6.355, 100.195]
Low*	650 (80.4)	483 (74)	167 (26)			
Health and health related variables						
**Health status of the HH**	808 (100)	629 (78)	179 (22)	.000	.	
Very poor^a^	50 (6.2)	48 (96)	2 (4)	.996	4.546	
Poor	166 (20.5)	145 (87)	21 (13)	.456	1.308	1.391 [.584, 3.315]
Medium*	270 (33.4)	227 (84)	43 (16)			
High	224 (27.8)	159 (71)	65 (29)	.012	.463	.381 [.179, .811]
Very high	98 (12.1)	50 (51)	48 (49)	.000	.197	.165 [.068, .402]
Member with chronic illness†	808 (100)	629 (78)	179 (22)			
Yes	61 (7.5)	57 (93)	4 (7)	.476	4.360	1.563 [.458, 5.337]
No*	747 (92.5)	572 (77)	175 (23)			
Illness in prev 3 months†	808 (100)	629 (78)	179 (22)			
No*	558 (69.0)	404 (72)	154 (28)			
Yes	250 (31.0)	225 (90)	25 (10)	.817	3.431	1.182 [.287, 4.869]
Seeking medical treatment†	808 (100)	629 (78)	179 (22)			
Yes*	231(28.6)	212 (92)	19 (8)			
No	577 (71.4)	417 (72)	160 (28)	.231	0.194	6.338 [.308, 130.434]
Borrow for treatment	808 (100)	629 (78)	179 (22)			
No*	530 (65.6)	381 (72)	149 (28)			
Yes	278 (34.4)	248 (89)	30 (11)	.004	3.233	2.836 [1.403, 5.730]
Time to HF (in minutes)	808 (100)	629 (78)	179 (22)	.001	.991	.983 [.973, .992]
Constant				.340		2.355

Note: *reference categories with highest frequency †variables which were significant in bivariate analysis but removed when the confounders are controlled in the multivariate logistic regression.

^a^Wildly improbable odds ratio.

Socioeconomic statuses of the respondents (educational status, wealth index and annual incomes) had also statistically significant associations with the households’ decision in WTJ the CBHIS. Respondents who had no education were about 3 times more likely to join the scheme than those who completed grade 1–8 (95% CI of AOR: 1.355, 7.143). Households who were in the highest wealth quintile were more than 4 times more likely to join the scheme than those who were in the second wealth quintile (95% CI of AOR: 1.626, 10.931). in the same manner, households with annual income 1100–4300 birrs were 2.105 times more likely to join the scheme than whose income was less than 4300 birrs [95% CI of AOR: 1.215, 3.650] (Table 4).

Participation in iddirs, number of iddirs the households participate in and amount of money the households contribute for iddir were not statistically significant in multivariate analyses. But the variables which measure both individual level and community level social capitals were positively associated with WTJ the CBHIS. Households with low individual level horizontal trust level were 93.6% less likely to join CBHIS than middle level ones (95% CI of AOR: .025, .165). Households of high individual level reciprocity were about 5 times more likely to join the scheme than those in middle level (95% CI AOR: 1.362, 18.052). Community level horizontal trust was strong positive predictor for WTJ community based health insurance (AOR = 25.2, 95% CI AOR: 6.355, 100.195). But community level reciprocity had no association in the decision of the household to join CBHIS (Table 4).

In case of health related variables, only self-reported health status of the household, borrowing money for covering treatments, and distance of the house to nearby health care facility were found to be significant predictors for the households’ WTJ decisions. Self-reported health status had negative association with the households’ WTJ. Borrowing money for health care payment was positively associated with WTJ. Households which borrowed money were about 3 times more likely to join the scheme than those who did not borrow (95% CI of AOR: 1.403, 5.730). Distance of the health facility to the home of the household, as measured by time taken to arrive at the nearby HF (HFs, here refer to hospital, public health center or private clinics), was negatively associated with WTJ. The probability of joining the scheme decreases by 1.6% as the time taken to reach the HFs increases by one minute. (95% CI of AOR: .973, .992) (Table 4).

### Discussion

After presenting the scenario of community based health insurance scheme, the respondents were asked whether they were willing to join the scheme. Presenting scenarios simplifies understanding of hypothetical markets such as community- based health insurance schemes which is new concept in the district [18].

Among 808 participants 629 (77.85%) responded that they would enroll in the scheme. Depending on the premium set, the actual enrollment could be lower. For example, if the premium is set to be 162.61 ETB (8.9 US$), only 50% of the households who are WTJ will enrol in the scheme. This translates the WTJ to be 38%. There are also other factors which may lower the actual WTJ [21].

But, this initial figure is greater than findings from Edo state of Nigeria (60%) [22]. The discrepancy may be attributed to the scenario employed in this study which was not used in the previous study. Various studies indicate that presenting scenarios about hypothetical markets such as health insurance schemes provides relatively accurate estimates. The difference may also be because of differences in the study areas. Obviously, people’s utilities in most aspects differ in different geographic regions.

In the current study, among the ill 231 (92.4%) had sought treatment for the illnesses they experienced, and 219 (94.8%) got treatment. This situation is not concurrence with the low access to health in Ethiopia [2]. This indicates that the health seeking behavior of the people in the current study area is better than the broader national instance.

The potential WTJ in the current study area also exceeds that in Ecuador, which is 69% [13]. This may be due to differences in study areas and demographic situations of the source population. The population in El Páramo Region of Ecuador, where the previous study was conducted, lacks official governance and the estimate number of the people is smaller than those in the current study. This situation led to small number of study participants which possibly yielded less precise estimate of WTJ in the El Páramo than the current findings.

The current finding is less than that found in 2004 in Ethiopia, in which the probability of WTJ the scheme was 94.7% [23]. The reason may be attributed to differences in the study areas and time of study. The current finding is almost similar to that conducted in Jimma town in 2009, in which the probability to join iddir-based health insurances were 76.5% [16].

The number of total family size, housewives (in comparison to farmers), participation in iddirs, amount contributed to iddirs monthly, individual social capital and community level horizontal trust had positive associations with the probability of WTJ the CBHIS. These findings are similar with those found in South Africa [24], Lao PDR [25], Nigeria [22] and rural areas of China [26,27].

One interesting finding in this study is that age of the respondent is negatively associated with WTJ of the households. This finding is inconcurrent with other findings [28-33]. The potential reason for such variation is pertinent to the benefit package of the proposed health insurance scheme in Ethiopia. Unlike health insurance schemes in many countries, the Ethiopian community-based health insurance scheme benefit package covers only the members of the households whose age is less than 18 years. As the age of the respondent in this study (mainly the head of the household) increases the probability of having family members who are eligible for the benefit package of the scheme is lower than the younger counterpart. Consequently the utility of joining the community-based health insurance scheme decreases. Such decision is in line with economic theories.

Few health related variables, such as seeking treatment during illnesses intrude, borrowing money for covering healthcare costs, which had no associations with probability of enrolling in iddir based health insurances in Jimma [16], had positive and significant associations with the outcome variable in the current study area. This discrepancy may be attributed to the differences in the study areas.

## Conclusion

In Debub Bench District If CBHIS commences about 78% of the households would enroll in the scheme. In the scheme the farmers, the married households, the younger, Bench ethnic groups and illiterate are more likely to enroll the schemes. The frequency of these population groups in the district are shown to be the majority. Consequently, acceptance of the scheme can be considered high. Therefore, initiating the scheme will be beneficial in the district.

## Abbreviations

AOR: Adjusted odds ratio; CBHI: Community-based health insurance; CBHIS: Community-based health insurance scheme; CHI: Community health insurance; ETB: Ethiopian Birr; IBHIS: Iddir-based health insurance scheme; SHI: Social health insurance; SNNPR: Southern Nations, Nationalities and People’s Region; WTJ: Willingness to join; WTP: Willingness to pay.

## Competing interests

We declare that we have no any competing interests.

## Authors’ contributions

ML was the principal investigator and wrote the paper. SO and BM made substantial contributions to the analysis and interpretation of the data. They also reviewed the first and second drafts. All authors read and approved the final manuscript.

## Pre-publication history

The pre-publication history for this paper can be accessed here:

http://www.biomedcentral.com/1471-2458/14/591/prepub

## Supplementary Material

Additional file 1 The questionnaire and community-based scenario used in the study. Click here for file
