# Supplementary material for: Willingness to join community-based health insurance among rural households of Debub Bench District, Bench Maji Zone, Southwest Ethiopia
Source: BMC Public Health. 2014 Jun 11;14:591. doi: 10.1186/1471-2458-14-591 (PMC4074337; doi:10.1186/1471-2458-14-591)
Supplement: Additional file 1 — The questionnaire and community-based scenario used in the study. [file 1471-2458-14-591-S1.pdf]

. QUESTIONNAIRE USED IN THE STUDY

Questionnaire for A Study on Willingness to Join and Pay For CBHI Among Households in Rural Areas of Debub Bench District, Bench Maji Zone, Southwest Ethiopia, 2013.

Hello, my name is ______________, I am one of the data collectors in this study. The study is intended to assess the demand and willingness to pay for the newly proposed community-based health insurance. To attain this purpose, your honest and genuine participation by responding to the questions prepared is very important and highly appreciated.

Confidentiality and consent

We would like you to answer some personal questions. Your answers are completely confidential and participation is voluntary. No one will be told what you said in connection to your name. You don't have to answer any question if you do not want to and you can stop the interview at any time.

However your honest answer to these questions will help us to better understand the situation and will contribute to improve the health status of the community by identifying the demand and ability to pay for CBHI and presenting to the policy makers so as to adjust the schemes with the local context.

We would greatly appreciate your help in participating in this study, would you be willing to participate?

☐ Yes, proceed

☐ No, good bye. Thank you for your cooperation!!!

____

(Signature of the interviewer certifying that respondent has given informed consent verbally).

QUESTIONNAIRE FOR A RESEARCH ON WILLINGNESS TO JOIN AND WILLINGNESS TO PAY FOR COMMUNITY-BASED HEALTH INSURANCE AMONG PEOPLE LIVING IN RURAL AREAS IN DEBUB BENCH DISTRICT, BENCH MAJI ZONE, SOUTHWEST ETHIOPIA, 2013.

*Questionnaire No*____

*Interviewer's Name*____

*Supervisor's Name*____

*Date of interview: dd*__________*mm*___________*yyyy*___________

*Interview times taken: started*____________*ended*________________

PART 1- HOUSEHOLD (SOCIO-DEMOGRAPHIC) CHARACTERISTICS			
Kebele_________________________________<br>House number________________________			
Q.	QUESTION	RESPONSE CODE (CHECK THE APPROPRIATE OPTION OR ENTER NUMBERS)	SKIP
100	What is the sex of the respondent?	1. Male ☐<br>2. Female ☐	
101	What is the age of the respondent?	Age in years (18-99) ☐☐ (write "99+", if >99 years)	
102	What is the relation of the respondent to the household?	1. Head ☐<br>2. Spouse ☐<br>3. Child ☐<br>4. Other (parent, sibling, child-in-law) ________	
103	What is the religion of the respondent?	1. Protestant ☐<br>2. Orthodox ☐<br>3. Muslim ☐<br>4. Other (specify)______________________	
104	Marital Status of the respondent	1. Monogamous /monandrous ☐<br>2. Polygamous/ polyandrous ☐<br>3. Single ☐<br>4. Widowed ☐<br>5. Divorced ☐	
105	Occupation of the respondent	1. Farmer ☐<br>2. Housewife ☐<br>3. Merchant ☐<br>4. Laborer ☐<br>5. Government ☐<br>6. Student ☐<br>7. Other(specify)_________	

106	Ethnicity of the respondent	1. Bench ☐<br>2. Me'enit ☐<br>3. Kaffa ☐<br>4. Amhara ☐<br>5. Tigre ☐<br>6. Other(specify)_________	
107	What is the educational status of the respondent	1. Illiterate ☐<br>2. Read and write ☐<br>3. Grade1-8 ☐<br>4. Secondary school ☐<br>5. Tertiary school ☐	
108	What is the size of the family of the household?	0-4 year old: male ☐ female ☐<br>5- 18 years old: male ☐ female ☐<br>19-64 years old: males ☐ females ☐<br>65^{+} years old: males ☐ females ☐<br>Total: males ☐☐; female ☐☐	
109	How many pregnant women are there in the household?	Number of pregnant women ☐	
110	Do you participate in *iddirs*?	1. Yes ☐<br>2. No ☐	If "no", skip to Q 211.
111	How many iddirs do you participate in?	Number ☐	
112	How much do you contribute for all iddirs you participate in per month?	Amount in Birr ☐☐☐☐	
PART 2: SOCIAL CAPITAL QUESTIONS			
2.1. Questions to assess Horizontal 'Trust'			
211	Generally, do you agree or disagree that most villagers of the village can be trusted?	1. Strongly disagree ☐<br>2. Disagree ☐<br>3. Neutral ☐<br>4. Agree ☐<br>5. Strongly agree ☐	
212	Do you agree that most villagers of the village would try to take advantage of you to achieve their own goals if they got a chance?	1. Strongly agree ☐<br>2. Agree ☐<br>3. Neutral ☐<br>4. Disagree ☐<br>5. Strongly disagree ☐	

213	Do you agree that most villagers would return what they pick up to the original owner?	1. Strongly disagree ☐<br>2. Disagree ☐<br>3. Neutral ☐<br>4. Agree ☐<br>5. Strongly agree ☐	
214	Do you agree most of your neighbors can be trusted?	1. Strongly disagre☐<br>2. Disagree ☐<br>3. Neutral ☐<br>4. Agree ☐<br>5. Strongly agree☐	
215	Do you agree the village leaders can be trusted?	1. Strongly disagree ☐<br>2. Disagree ☐<br>3. Neutral ☐<br>4. Agree ☐<br>5. Strongly agree ☐	
2.2. Questions to assess reciprocity			
221	Do you agree villagers concern issues that not only relate to themselves, but also relate to others?	1. Strongly disagree☐<br>2. Disagree ☐<br>3. Neutral ☐<br>4. Agree ☐<br>5. Strongly agree ☐	
222	Do you agree villagers will provide help if someone really needs it?	1. Strongly disagree☐<br>2. Disagree ☐<br>3. Neutral ☐<br>4. Agree ☐<br>5.Strongly agree ☐	
223	Do you agree that you would lend money to your neighbor if he/she needs it to see a doctor?	1. Strongly disagree ☐<br>2. Disagree ☐<br>3. Neutral ☐<br>4. Agree ☐<br>5.Stronglyagree ☐	
224	Do you agree that, if your village were a large family, you would be a member in this family?	1. Strongly disagree ☐<br>2. Disagree ☐<br>3. Neutral ☐<br>4. Agree ☐<br>5. Strongly agree ☐	
225	Do you agree that you would like to support a project that might not benefit you most, but benefit other villagers?	1. Strongly disagree ☐<br>2. Disagree ☐<br>3. Neutral ☐<br>4. Agree ☐<br>5. Strongly agree ☐	
PART 3- Health and health related questions			
300	How do you rate the health status of your family?	1. Very poor ☐<br>2. Poor ☐<br>3. Medium ☐<br>4. Good ☐<br>5. Very good☐	
301	Do you or other member of the household have chronic illness and/or	1. Yes ☐<br>2. No ☐	

	disability?		
302	Have any member of the family encountered any illness during the past 3 months?	1. Yes ☐<br>2. No ☐	If "no", skip to Q 315.
303	How many of the members were ill?	The number of the ill ☐	
304	Did you seek medical treatment for the recent episode?	1. Yes ☐<br>2. No ☐	If "no", skip to Q 315
305	Did you get treatment?	1. Yes ☐<br>2. No ☐	If "no", skip to Q 308.
306	Where did you get treatment?	1. Self-treatment ☐<br>2. Local drug vender ☐<br>3. Private Heath Facility ☐<br>4. Public health center ☐<br>5. Public hospital ☐<br>6. Traditional healer ☐<br>7. Other (specify)_________________	
307	Why did you go there?	1. The HF was physically accessible ☐<br>2. The HF was not expensive ☐<br>3. The health facility not too crowded ☐<br>4. The health service was courteous ☐<br>5. The health service was efficacious/ effective ☐<br>6. Other (specify)_______________	
308	Why did not you get treatment?	1. Considering the illness is self-limiting ☐<br>2. No enough money ☐<br>3. Didn't know anywhere to go ☐<br>4. Didn't have time to go to health facility ☐<br>5. Too far to go health facility ☐<br>6. Other (specify) _______________<br>99. unknown	Go to Q 315
309	How much was total health care cost of the household for the treatment in the last 3 months?	Amount in Birrs ☐☐☐☐	
310	Who covered the health care cost?	1. Self ☐<br>2. Government/free ☐<br>3. Community ☐<br>4. Other (specify)________________	

311	How was your satisfaction with health care service and costs?	1. Very dissatisfied ☐<br>2. Dissatisfied ☐<br>3. Neutral ☐<br>4. Satisfied ☐<br>5. Very satisfied ☐	
312	How did you perceive quality of the health care service in this area?	1. Very low ☐<br>2. Low ☐<br>3. Neutral ☐<br>4. High ☐<br>5. Very high ☐	
313	How did you see finding money to pay for the health care?	1. Very difficult ☐<br>2. Difficult ☐<br>3. Not difficult ☐	If "3", skip to Q 315
314	If paying for a medical expense was difficult, how did you get it?	1. Drew from the savings ☐<br>2. Borrow from someone ☐<br>3. Assisted by relatives ☐<br>4. Undertaken extra work ☐<br>5. Sell capital assets such as cows ☐<br>6. Cut back on other things, food, drink, cloth etc ☐<br>7. Others (specify) _______________	
315	Did you borrow any money from relatives or other people to cover medical costs within the last year?	1. Yes ☐<br>2. No ☐	If "no", skip to Q 317
316	How much did you borrow?	Amount borrowed in Birrs ☐☐☐☐	
317	What is the nearest conventional health institution to your home	1. Health center ☐<br>2. Clinic (Private) ☐ 3. Hospital (Gov) ☐	
319	How long does it take to reach the nearby HF from your home?	Time in minutes ☐☐☐	
PART-4: INCOME AND WEALTH INDEX QUESTIONS			
400	Approximately, how much of these products did your household produced and sold during the last 1 year?	1. Coffee (in Birr) ☐☐☐☐☐<br>2. Khat sold in Birr ☐☐☐☐☐<br>3. Maize (in Birr) ☐☐☐☐☐<br>4. Cassava(in quintals) ☐☐☐☐<br>5. Others (specify) ______________	
401	How many of these animals do this household own?	1. Milk cows, oxen or bulls? ☐☐<br>2. Goats? ☐☐<br>3. Sheep? ☐☐	

		4. Chickens? ☐☐<br>5. Beehives ☐☐<br>6. Other(specify) ______________	
402	Does your household have?		
	a) Functioning radio/tape	1. Yes ☐ 2. No ☐	
	b) Horse/mule /Donkey	1. Yes ☐ 2. No ☐	
	c) Cotton/sponge/spring mattress?	1. Yes ☐ 2. No ☐	
	d) Bed	1. Yes ☐ 2. No ☐	
403	What kind of latrine does your family have?	1. None ☐<br>2. VIP ☐<br>3. Traditional latrine ☐<br>4. Other (specify)___________	
404	What is the type of roof of the house?	1. Corrugated sheet ☐<br>2. Thatch roof ☐<br>3. Other (specify)________________	
405	How many rooms are used by this household for sleeping only?	Number of rooms ☐	
406	Do you have kitchen	1. Yes ☐ 2. No ☐	
407	Do you have separate rooms for cattle?	1. Yes ☐ 2. No ☐	
408	What is the wall of your residence house made of?	1. Wooden structure ☐<br>2. Mud ☐<br>3. Other (specify)___________	
409	What is the total farm size holding of the household in Hectares?	Size in hectares ☐☐☐	

PART 5: CBHI SCENARIO

INTRODUCTION

Sickness needs to be treated immediately and it is not possible to wait. If you don't have the money available, then you will need to borrow it from your neighbor or sell your sheep or chickens. While you run around trying to get the money together, the sick person suffers. And many times it happens that you come back with the money only to find out that the sick person has died.

In order to solve this financial problem, The Federal Democratic Republic of Ethiopia, Ministry of Health is planning to set community-based health insurance in your community, if the community members want to be included in such programs. If you join the insurance and pay an annual premium, you don't need to pay for the following health services provided in your district area for a period of one year for your family members (the head of the household, the spouse and children age of less than 18years).

BENEFITS PACKAGE

Drugs: all essential and generic drugs which you already buy in your pharmacy, either at the health center level or at the district hospital level. The insurance may pay for drugs sold in accredited private drugstores.

Laboratory tests: all costs of laboratory tests that have been prescribed by the public health agent are covered if they are being carried out in accredited public or private health facilities.
Inpatient stays: when you are hospitalized, the insurance will cover the expenses. Urgent transport by ambulance from your village to the primary hospital in the locality will also be covered. Surgery: general surgery and delivery complications, extraction of teeth and circumcision are included. But surgeries for cosmetics purposes such as plastic surgery are not included. X-rays will be covered if the doctor thinks it is necessary.
Organization of the insurance scheme committee selected from your kebele workers and the health extension workers will manage the scheme. Some of the premiums will be kept at kebele level by the committee to cover the provision of drugs. The premiums will be kept in a bank. The committee chair and a treasurer have a right to withdraw money from the bank and to pay health facilities. An annual audit will ensure that funds are used rationally. The committee will give a financial report of the scheme to local government every year.

ENROLMENT AND PAYMENT

You need to pay an annual premium for joining the insurance and 5.00 ETB for registration Credit is not allowed. After you pay the premiums, then you can enjoy the benefits of insurance after 3 months. If you don't pay the premiums, you have to pay for all service 'out-of-pocket'. For example, if you suffer from typhoid, you have to pay for the treatment and laboratory diagnosis services.

REIMBURSEMENT PROCEDURE

Insured patients don't need pay money to see a doctor for the services that are covered by the insurance. The money will be paid by the committee. But patients will have to pay for services not covered by the insurance.

PART 6: WILLINGNESS TO JOIN THE CBHI

600	Based on the above scenario, will you join the CBHIS if it is established in your community?	1. Yes ☐<br>2. No ☐<br>3. Undecided ☐	If "no", skip to Q 602
601	If yes, why do you join the CBHIS	1. It provides free access to medical care ☐<br>2. To help others ☐<br>3. For security and peace of mind in times of ill-health ☐<br>4. Facing health problem frequently ☐<br>5. Other(specify)_______	
602	Why will you not join the scheme?	1. I do not have enough money to pay ☐<br>2. Do not need health insurance ☐<br>3. OOP charge is better ☐<br>4. Lack of trust in government programmes ☐<br>5. Lack of trust in insurance practitioners ☐<br>6. Lack of functional HF in my village ☐<br>7. H/insurance is a confusing scheme ☐<br>8. No qualified h/personnel in the HC ☐<br>9. Contributing money for sickness in advance is a taboo ☐<br>10. Scopes of illnesses covered by the scheme is limited ☐	Stop the interview here

		11. Others (specify) _____________	
PART 7: WILLINGNESS TO PAY			
700	Which initial bid have you taken	1. 50 ETB☐ 4. 300 ETB☐<br>2. 100 ETB☐ 5. 400 ETB☐<br>3. 180 ETB☐ 6. 500 ETB☐	
702	If you join the scheme will you pay the specified amount per year as a premium?	1. Yes ☐<br>2. No ☐	If "no", skip to Q 704
702	Will you pay if the premium is double of the specified Birr amount per year per household?	1. Yes ☐<br>2. No ☐	If "no", skip to Q 707.
703	Maximum how much shall you pay per year per household?	Specify the amount in Birr☐☐☐☐	Go to Q 707
704	If'no" for Q 700, will you pay (half of the initial bid)/year/HH?	1. Yes ☐<br>2. No ☐	If "yes", skip to Q 707
705	If "no" for Q704, maximum how much shall you pay per year per household?	Specify the amount in birr☐☐☐☐	Go to Q 706, if answer = 0 birr; go to 707 if >0 birr.
706	If the answer is 0.00 birr for Q 705, why is your household not willing to pay for the scheme?	1. I doubt the management of the fund☐<br>2. It is the responsibility of the government to pay for such a programme ☐<br>3. Because of lack of money ☐<br>4. Other members of the society should pay for the programme ☐<br>5. Other (specify)________________	Stop the interview here
707	How frequently do you want to pay the yearly premium?	1. Annual flat rate ☐<br>2. Bi-annual flat-rate ☐<br>3. Quarterly a year flat-rate ☐<br>4. Monthly ☐<br>5. Other (specify)________________	
